# A prospective multicentre diagnostic accuracy study for the Truenat tuberculosis assays

**DOI:** 10.1183/13993003.00526-2021

**Published:** 2021-11-04

**Authors:** Adam Penn-Nicholson, Sivaramakrishnan N. Gomathi, Cesar Ugarte-Gil, Abyot Meaza, Evelyn Lavu, Pranav Patel, Bandana Choudhury, Camilla Rodrigues, Sarabjit Chadha, Mubin Kazi, Aurélien Macé, Pamela Nabeta, Catharina Boehme, Raman R. Gangakhedkar, Sanjay Sarin, Ephrem Tesfaye, Eduardo Gotuzzo, Philipp du Cros, Srikanth Tripathy, Morten Ruhwald, Manjula Singh, Claudia M. Denkinger, Samuel G. Schumacher, the Truenat Trial Consortium

**Affiliations:** 1 FIND, Geneva, Switzerland; 2 National Institute for Research in Tuberculosis, Chennai, India; 3 Instituto de Medicina Tropical Alexander von Humboldt, Lima, Peru; 4 School of Medicine, Universidad Peruana Cayetano Heredia, Lima, Peru; 5 Ethiopian Public Health Institute, Addis Ababa, Ethiopia; 6 Central Public Health Laboratory, Port Moresby, Papua New Guinea; 7 State TB Demonstration and Training Centre, Ahmedabad, India; 8 Intermediate Reference Laboratory, Guwahati, India; 9 PD Hinduja Hospital, Mumbai, India; 10 FIND – India, New Delhi, India; 11 Indian Council of Medical Research, New Delhi, India; 12 Burnet Institute, Melbourne, Australia; 13 Division of Tropical Medicine, Center of Infectious Disease, University Hospital Heidelberg, Heidelberg, Germany; 14 These authors contributed equally; 15 The members of the Truenat Trial Consortium are listed in the Acknowledgements

## Abstract

**Background:**

Bringing reliable and accurate tuberculosis (TB) diagnosis closer to patients is a key priority for global TB control. Molbio Diagnostics have developed the Truenat point-of-care molecular assays for detection of TB and rifampicin (RIF) resistance.

**Methods:**

We conducted a prospective multicentre diagnostic accuracy study at 19 primary healthcare centres and seven reference laboratories in Peru, India, Ethiopia and Papua New Guinea to estimate the diagnostic accuracy of the point-of-care Truenat MTB, MTB Plus and MTB-RIF Dx assays for pulmonary TB using culture and phenotypic drug susceptibility testing as the reference standard, compared with Xpert MTB/RIF or Ultra.

**Results:**

Of 1807 enrolled participants with TB signs/symptoms, 24% were culture-positive for *Mycobacterium tuberculosis*, of which 15% were RIF-resistant. In microscopy centres, the pooled sensitivity of Truenat MTB and Truenat MTB Plus was 73% (95% CI 67–78%) and 80% (95% CI 75–84%), respectively. Among smear-negative specimens, sensitivities were 36% (95% CI 27–47%) and 47% (95% CI 37–58%), respectively. Sensitivity of Truenat MTB-RIF was 84% (95% CI 62–95%). Truenat assays showed high specificity. Head-to-head comparison in the central reference laboratories suggested that the Truenat assays have similar performance to Xpert MTB/RIF.

**Conclusion:**

We found the performance of Molbio's Truenat MTB, MTB Plus and MTB-RIF Dx assays to be comparable to that of the Xpert MTB/RIF assay. Performing the Truenat tests in primary healthcare centres with very limited infrastructure was feasible. These data supported the development of a World Health Organization policy recommendation of the Molbio assays.

## Abstract 2

**Diagnostic performance of point-of-care Truenat assays in primary healthcare centres is comparable to that of Xpert MTB/RIF placed in reference laboratories. The WHO now recommends Truenat as an initial test for detection of TB and RIF resistance.** https://bit.ly/31Wj3S6

## Introduction

Effective control of the tuberculosis (TB) epidemic requires rapid diagnosis and initiation of appropriate treatment. However, of the estimated 10 million new TB cases in 2019, 2.9 million cases went undiagnosed [1]. Only 61% of bacteriologically confirmed TB cases were tested for rifampicin (RIF) resistance [1]. Conventional culture and drug susceptibility testing (DST) methods rely on the slow growth of *Mycobacterium tuberculosis* in solid or liquid media, which can take weeks to months to yield results [1], and can lead to prolonged periods of ineffective therapy and ongoing disease transmission. Furthermore, many countries with high TB burdens lack the resources to establish the stringent laboratory conditions needed for these growth-based methods and must rely upon sputum smear microscopy tests, which, on average, detect only 45% of TB infections [2].

Bringing rapid and accurate TB and drug resistance diagnostics closer to patients is a key priority for TB control, particularly to reach patients in low-resource settings and avoid existing high rates of pre-treatment loss to follow-up [3]. This requires robust point-of-care diagnostic tests that are easily implementable at lower levels of the healthcare system.

Xpert MTB/RIF and Xpert MTB/RIF Ultra (“Ultra”) have revolutionised the diagnosis of both TB and RIF resistance [4, 5], with Xpert MTB/RIF demonstrating pooled sensitivity of 85% (95% CI 82–88%) and specificity of 98% (95% CI 94–97%), and Ultra providing slightly higher sensitivity of 88% (95% CI 85–91%) and slightly lower specificity of 96% (95% CI 94–97%) in a recent systematic review [6]. However, these tests, run on GeneXpert instruments (Cepheid, Sunnyvale, CA, USA), require a temperature-controlled environment, a stable power supply and are susceptible to dust [5, 7–10], limiting operation to district/subdistrict hospital settings. A novel point-of-care, cost-effective assay with higher performance and/or a robust, battery-operated assay with minimal operational requirements could provide a viable alternative to Xpert and drive greater access for TB testing. Molbio Diagnostics (Bangalore, India) developed three assays that utilise chip-based real-time micro PCR: two for detection of *M. tuberculosis* (the Truenat MTB assay (including the *nrdB* single copy target) and the MTB Plus assay (including *nrdZ* and multicopy *IS6110* targets)) and one for the detection of RIF resistance (the MTB-RIF Dx reflex assay targeting the *rpoB* gene) [11, 12]. These assays can be run from the same DNA eluate [13–16], obtained from the automated bead-based Trueprep DNA extraction device that uses a universal cartridge-based system to extract DNA from 0.5 mL of sputum in <20 min. The DNA eluate is loaded onto the chip-based Truelab micro PCR device to detect the presence of *M. tuberculosis* DNA in ∼40 min. If *M. tuberculosis* is detected, the Truenat MTB-RIF Dx reflex test can similarly be run in the Truelab machine using the same DNA eluate. Both the Trueprep and Truelab devices are portable, battery operated, and can function at up to 40°C ambient temperature and up to 80% relative humidity [17, 18].

Here, we report results from a multicentre diagnostic accuracy study of the Truenat MTB, MTB Plus and MTB-RIF Dx assays, in which we assessed performance at the primary healthcare centre level against culture and phenotypic DST as a reference standard, and compared against the performance of Xpert MTB/RIF, Ultra and the Truenat assays conducted at centralised reference laboratories.

## Methods

### Study design

This prospective, multicentre diagnostic accuracy study of the performance of the Truenat TB assays was conducted in 19 clinical sites (with attached microscopy centres) and seven reference laboratories across Ethiopia, India, Papua New Guinea and Peru (supplementary table S1). The study population comprised adult men and women presenting to clinics with symptoms suggestive of pulmonary TB disease (supplementary table S2). Participants were recruited sequentially at each clinic or through neighbouring satellite clinics and enrolled once informed consent was obtained into either a “Case Detection Group” or a “Drug-Resistant Risk Group” (supplementary material).

The study was conducted in accordance with the 1964 Helsinki Declaration and its subsequent amendments, and approved by the relevant institutional review boards and independent ethics committees. All participants provided informed consent, either written, or if illiterate, as a thumbprint on the consent form signed and dated by an impartial witness. This study is registered at ClinicalTrials.gov with identifier number NCT03712709.

### Procedures

**FIGURE 1:**
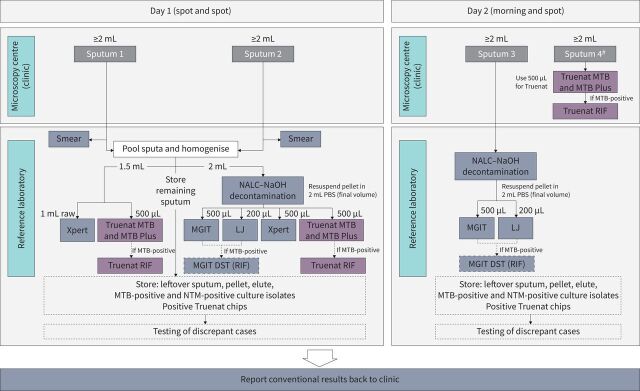
Specimen flow at enrolment. NALC–NaOH: *N*-acetyl-l-cysteine–sodium hydroxide; MGIT: Mycobacterial Growth Indicator Tube; LJ: Löwenstein–Jensen; MTB: *Mycobacterium tuberculosis*; DST: drug susceptibility testing; RIF: rifampicin; NTM: nontuberculous mycobacteria. ^#^: sputum 4 was not collected at PD Hinduja Hospital or in Papua New Guinea. All sites performed Xpert MTB/RIF except Peru, which performed Xpert MTB/RIF Ultra. As Truenat assays are not indicated for decontaminated sputum sediments and do not contribute to our study objective, test results are not presented within this article, but are available upon request.

Participants enrolled at primary healthcare centre clinics were asked to provide three sputum specimens for reference laboratory testing and an additional specimen for microscopy centre testing (figure 1 and supplementary material). Sputum specimens 1, 2 and 3 were transported to the centralised reference laboratory for culture, Xpert MTB/RIF or Ultra, Truenat and smear testing. Sputum specimen 4 remained at the attached microscopy centre for Truenat assay testing.

Laboratory testing was performed by index and reference standard tests (figure 1 and supplementary table S3). Quality-assured smear microscopy (predominantly Ziehl–Neelsen staining, although auramine-O fluorescence staining was used in PD Hinduja Hospital in India and both methods were used in Peru), liquid (Mycobacterial Growth Indicator Tube (MGIT)) and solid (Löwenstein–Jensen) culture, BACTEC MGIT 960 phenotypic DST, and speciation [19] were performed at the reference laboratories using two independent sputa per participant. All reference laboratories used Xpert MTB/RIF as the comparator due to Ultra availability issues at study initiation, except the reference laboratory in Peru, which only used Ultra. Truenat testing was done either in the reference laboratory (day 1 sputa) or the microscopy centre (day 2 sputa) and was performed as per the manufacturer's recommendations [20–22]. Truenat test results were not shared with clinical staff and did not influence patient treatment options.

### Statistics and analysis

A sample size of 1666 participants was selected to allow analysis of 80 (95% CI 55–77) smear-negative culture-positive TB cases across sites. Participants in the Case Detection Group were included in all analyses, whereas participants in the Drug-Resistant Risk Group were only included in analyses of RIF resistance detection. Analyses of the diagnostic accuracy of the Truenat index tests and comparator tests were conducted per case or per specimen in the Case Detection Group and reported as point estimates and 95% confidence intervals based on Wilson's score method. Subgroup analyses by site of testing (microscopy centre *versus* reference laboratory for Truenat), smear status, TB history and HIV status were performed. The study protocol and statistical analysis plan are available in the supplementary material. All statistical analysis was performed using R version 3.5.1 (www.r-project.org).

## Results

### Participant demographics

**FIGURE 2:**
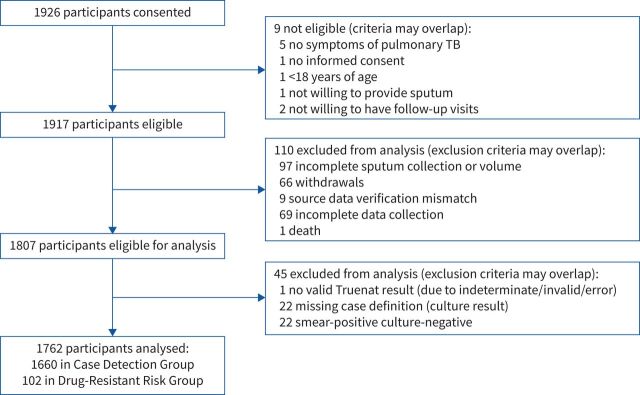
STARD (Standards for Reporting of Diagnostic Accuracy Studies) diagram showing the number of participants enrolled, excluded and with data analysed. TB: tuberculosis. Truenat nondeterminate results are excluded from the accuracy analyses but are reported separately.

Between March 2019 and February 2020, 1917 participants met the eligibility criteria for enrolment across the 19 study sites (figure 2). After excluding 155 participants due to incomplete data (missing culture or index test results), a total of 1762 participants remained for the analysis. Of the 1762 participants, 1660 (94%) were in the Case Detection Group for analysis of accuracy for *M. tuberculosis* detection and 102 (6%) already on treatment regimens at the time of enrolment met the criteria of the Drug-Resistant Risk Group. A total of 331 participants only had a sputum sample collected at the reference laboratory setting and not at the primary healthcare centre, and 21 of those participants did not have any available culture result.

Demographic and clinical characteristics of the enrolled participant population are shown in table 1. The median (range) age of participants was 41 (18–88) years, with women making up 43% of the total participant population. HIV results were only available for 51% (n=903) of the participants, for whom HIV prevalence was 5.3% (n=48), including 12 diagnosed with active TB. The prevalence of TB (based on culture as the reference standard) across all sites was 24%, with 22% in the Case Detection Group and 66% in the Drug-Resistant Risk Group. Among the 358 culture-positive participants in the Case Detection Group, 32% tested negative by smear microscopy on both specimens. The prevalence of RIF resistance in culture-positive participants, based on phenotypic DST results, was 15% in total (13% among new cases and 24% among participants in the Drug-Resistant Risk Group). PD Hinduja Hospital, a drug-resistant TB referral clinic, contributed 51% (32 out of 63) of all RIF-resistant cases diagnosed as part of the study and 31% (32 out of 102) of all enrolled participants at PD Hinduja Hospital were RIF-resistant.

**TABLE 1:** Demographic and clinical characteristics of enrolled participant population

	**All**	**India**				**Peru**	**Ethiopia**	**Papua New Guinea**
		**Hinduja**	**Guwahati**	**Chennai**	**Ahmedabad**			
**Participants**	1762	144	256	319	290	394	196	163
**Age years**	41 (18–88)	39 (18–86)	42 (18–82)	48 (19–83)	47 (19–85)	38 (19–88)	37 (18–81)	34 (18–78)
**Female**	43 (762/1762)	50 (71/144)	36 (91/256)	43 (136/319)	36 (103/290)	50 (196/394)	51 99/196	40 (66/163)
**HIV-infected** ^#^	5.32 (48/903)	1.61 (1/62)	0 (0/5)	0 0/313)	(1/165)	2.68 (7/261)	61 (28/46)	22 (11/51)
**Culture-positive**	24 (425/1762)	71 (102/144)	23 (59/256)	13 (40/319)	19 (55/290)	24 (96/394)	12 (24/196)	30 (49/163)
**Smear-negative culture-positive**	30 (128/425)	22 (22/102)	25 (15/59)	38 (15/40)	18 (10/55)	44 (42/96)	33 (8/24)	33 (16/49)
**DST RIF-resistant among culture-positive**	15 (63/425)	31 (32/102)	19 (11/59)	2.5 (1/40)	5.5 (3/55)	11 (11/96)	4.2 (1/24)	8.2 (4/49)
**Drug-Resistant Risk Group**	5.8 (102/1762)	67 (96/144)	0 (0/256)	0 (0/319)	1.0 (3/290)	0.8 (3/394)	0 (0/196)	0 (0/163)

Data are presented as N, median (minimum–maximum) or % (n/N). DST: drug susceptibility testing; RIF: rifampicin. ^#^: proportion of HIV infection was reported based on available test results.

### Diagnostic accuracy of the Truenat MTB detection assays

For specimens tested in the primary healthcare centres, 1356 participants in the Case Detection Group had valid Truenat results for both the MTB and MTB Plus assays and had valid culture results. Of these, 263 participants were culture-positive with *M. tuberculosis* complex identification; 177 were smear-positive culture-positive and 86 were smear-negative culture-positive.

For testing at primary healthcare centres, sensitivity was 73% (95% CI 67–78%) for Truenat MTB and 80% (95% CI 75–84%) for Truenat MTB Plus (table 2 and supplementary table S4). Specificity was 98% (95% CI 97–99%) and 96% (95% CI 95–97%) for Truenat MTB and MTB Plus, respectively. Sensitivity for smear-negative culture-positive participant specimens was 36% (95% CI 27–47%) for Truenat MTB and 47% (95% CI 36–57%) for Truenat MTB Plus (table 2). Comparison of the diagnostic accuracy of Truenat MTB and MTB Plus assays on the same sputum specimens in the primary healthcare centre showed higher sensitivity for Truenat MTB Plus than Truenat MTB (sensitivity difference +6.8%, 95% CI +3.5– +20%), with lower specificity (specificity difference −1.4%, 95% CI −2.5– −0.3%). There was no appreciable difference in accuracy for any Truenat assay run at the primary healthcare centres or reference laboratories (supplementary table S5). While sensitivity of the Truenat MTB assay was marginally lower in the primary healthcare centres (difference −5.4%, 95% CI −10– −1.2%), the small sample size, known heterogeneity across sputa collected on different days and lack of difference for the Truenat MTB Plus or MTB-RIF Dx assays suggest caution in interpretation. Additional subanalyses by TB history are reported in supplementary table S6.

**TABLE 2:** Performance of Truenat assays for tuberculosis and for rifampicin resistance detection at the primary healthcare centre (microscopy centre) and the reference laboratory

	**N**	**True positive**	**False positive**	**False negative**	**True negative**	**Sensitivity % (95% CI)**	**Sensitivity % smear-positive (95% CI)**	**Sensitivity % smear-negative (95% CI)**	**Specificity % (95% CI)**
**Microscopy centre sputum**									
Truenat MTB	1356	192	25	71	1068	73.0 (67.3–78.0)	91.0 (85.8–94.4) (n=177)	36.0 (26.7–46.6) (n=86)	97.7 (96.7–98.5)
Truenat MTB Plus	1356	210	40	53	1053	79.8 (74.6–84.2)	96.0 (92.1–98.1) (n=177)	46.5 (36.4–57.0) (n=86)	96.3 (95.1–97.3)
Truenat MTB-RIF Dx	190	16	9	3	162	84.2 (62.4–94.5)	87.5 (64.0–96.5) (n=16)	66.7 (20.8–93.8) (n=3)	94.7 (90.3–97.2)
**Reference laboratory sputum**									
Truenat MTB	1541	275	27	71	1168	79.5 (74.9–83.4)	95.8 (92.4–97.7) (n=236)	44.5 (35.6–53.9) (n=110)	97.7 (96.7–98.4)
Truenat MTB Plus	1541	295	51	51	1144	85.3 (81.1–88.6)	98.3 (95.7–99.3) (n=236)	57.3 (47.9–66.1) (n=110)	95.7 (94.4–96.7)
Truenat MTB-RIF Dx	332	44	9	8	271	84.6 (72.5–92.0)	86.7 (73.8–93.7) (n=45)	71.4 (35.9–91.8) (n=7)	96.8 (94.0–98.3)

Analysis of Truenat performance is shown on specimens tested at the microscopy centre and at the reference laboratory separately, with valid results available for both the Truenat MTB assay and the Truenat MTB Plus assay; denominators differ as two sites (PD Hinduja Hospital and Papua New Guinea) only had reference laboratory facilities available. Comparative performance of each assay performed on samples processed in the microscopy centre or the reference laboratory is shown in supplementary table S5.

### Diagnostic accuracy of the Truenat MTB RIF detection assay

DNA extracted from participant sputum with a positive result on either the Truenat MTB or MTB Plus assay was reflexed for subsequent testing on the Truenat MTB-RIF Dx assay. At the primary healthcare centre the Truenat MTB-RIF Dx assay had 84% (95% CI 62–95%) sensitivity and 95% (95% CI 90–97%) specificity for RIF resistance detection relative to RIF DST (table 2). The MTB-RIF Dx assay conducted on sputum in the reference laboratories had a sensitivity of 85% (95% CI 73–92%) and specificity of 97% (95% CI 94–98%) (table 2). There was no difference in performance of the Truenat MTB-RIF Dx assay run in the primary healthcare centres and the reference laboratories (supplementary table S5).

### Diagnostic accuracy of Truenat assays compared with Xpert MTB/RIF and Ultra

**FIGURE 3:**
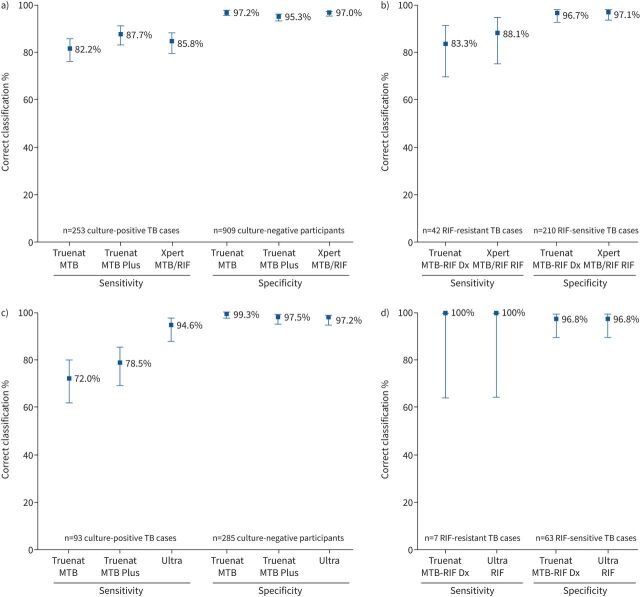
Performance of the Truenat, Xpert MTB/RIF and Ultra assays conducted at the reference laboratories. TB: tuberculosis; RIF: rifampicin. a) Performance of Truenat and Xpert MTB/RIF for TB detection (participants from Case Detection Group). b) Performance of Truenat and Xpert MTB/RIF for RIF resistance detection (all participants). c) Performance of Truenat and Ultra for TB detection (participants from Case Detection Group). d) Performance of Truenat and Ultra for RIF resistance detection (all participants).

To compare the performance of Truenat with Xpert MTB/RIF and Ultra, specimens received in the reference laboratory were split and tested side by side on Truenat and Xpert assays (Ultra was used instead of Xpert MTB/RIF in Peru). Among 1542 participants in the Case Detection Group with valid culture, Truenat and Xpert MTB/RIF or Ultra results, performance of Truenat MTB and MTB Plus was largely comparable to that of Xpert MTB/RIF (figure 3a). In sites where Xpert MTB/RIF was run on raw sputa, the sensitivities were 82% (95% CI 77–86%) for Truenat MTB, 88% (95% CI 83–91%) for Truenat MTB Plus and 86% (95% CI 81–90%) for Xpert MTB/RIF; respective specificities were 97% (95% CI 96–98%) for Truenat MTB, 95% (95% CI 94–97%) for Truenat MTB Plus and 97% (95% CI 97–98%) for Xpert MTB/RIF. In Peru, the only site where Ultra testing was performed, the sensitivities were 72% (95% CI 63–80%) for Truenat MTB, 79% (95% CI 70–86%) for Truenat MTB Plus and 95% (95% CI 88–98%) for Ultra; respective specificities were 99% (95% CI 98, 100) for Truenat MTB, 98% (95% CI 95–99%) for Truenat MTB Plus and 97% (95% CI 95–98%) for Ultra (figure 3a and c). There was no significant difference in performance of the Truenat assays compared with Xpert MTB/RIF, irrespective of smear status (supplementary table S6). In Peru, sensitivity was higher in Ultra than Truenat MTB (difference −23%, 95% CI −15– −32%) and MTB Plus (difference −16%, 95% CI −10– −25%) (supplementary table S7). Ultra and Truenat MTB specificities were comparable.

For the 252 individuals with valid Truenat TB detection and Xpert MTB/RIF results, the sensitivities of Truenat MTB-RIF Dx and Xpert MTB/RIF assays for RIF resistance detection were 83% (95% CI 70–92%) and 88% (95% CI 75–95%), respectively; specificities were 97% (95% CI 93–98%) for Truenat MTB-RIF Dx and 97% (95% CI 94–99%) for Xpert MTB/RIF (figure 3b). In Peru (the only site where Ultra was used), specimens from 70 participants were reflexed to Truenat MTB-RIF Dx testing, and sensitivity was 100% (95% CI 65–100%) and specificity was 97% (95% CI 89–99%) for both Truenat MTB-RIF Dx and Ultra tests (figure 3d). There was no difference in performance of Truenat MTB-RIF Dx against either Xpert MTB/RIF or Ultra (supplementary tables S6 and S7).

### Nondeterminate results for Truenat, Xpert MTB/RIF and Ultra assays

The proportion of initial Trueprep nondeterminate results was 2.4% (113 out of 4731) (supplementary table S9). A single round of repeat testing, where possible, resolved results for 88% (98 out of 111) of the specimens that failed on the initial test. Initial test nondeterminate proportions for the Truenat MTB and MTB Plus chip were 6.2% (293 out of 4720) and 9.2% (434 out of 4720), respectively. Of the tests that failed, 21% (62 out of 293) and 37% (159 out of 432) remained nondeterminate upon repeat testing. Comparatively, the nondeterminate rate of Xpert MTB/RIF was 2.6% (65 out of 2522), with no failures observed for Ultra (0 out of 786).

The nondeterminate rate for the Truenat MTB-RIF Dx assay initial test was 23% (232 out of 1042), of which 73% (157 out of 216) did not resolve where repeat testing was possible. The nondeterminate rate increased with low bacterial load in the specimen: the proportion of nondeterminate Truenat MTB-RIF Dx results was 6.7% (58 out of 886) if reflexed from a Truenat MTB-positive result *versus* 72% (26 out of 36) if reflexed from a specimen that was Truenat MTB-negative but Truenat MTB Plus-positive (supplementary table S10).

## Discussion

This multicentre diagnostic accuracy study indicates that the rapid molecular Truenat assays have overall comparable performance characteristics to Xpert and could be considered as initial tests for the diagnosis of TB and detection of RIF resistance in primary healthcare facilities [23]. The specificity of the assays in the primary healthcare centre was equivalent to that seen in the reference laboratory, despite the open nature of the assay.

For TB detection, the low sensitivity of the Truenat MTB and MTB Plus assays in smear-negative participants was unexpected. However, the head-to-head comparison to Xpert MTB/RIF showed similarly low sensitivity for Xpert MTB/RIF, suggesting that suboptimal performance was due to a challenging patient spectrum, rather than poor assay performance. In Peru, the higher sensitivity of Ultra may be related to the inclusion of the *IS1081* target in Ultra, which is missing in the Truenat assays, although interpretation of these results should consider the limited sample size in Peru. The known heterogeneity in performance frequently seen across different Xpert MTB/RIF and Ultra accuracy studies may also reflect population or patient spectrum specific differences [6].

The low incidence of nondeterminate Truenat MTB and MTB Plus results provides reassurance that the assays can be performed in primary healthcare settings. These findings are largely in line with those for Xpert nondeterminate results and reflect results seen in early Xpert evaluation studies [24, 25], although unlike Xpert, the Truenat assays were conducted in primary healthcare facilities. However, the proportion of nondeterminate results for Truenat MTB-RIF Dx was high: 20% of all initial tests, with 73% of these remaining unresolved upon re-testing. The finding that the Truenat MTB-RIF Dx assay nondeterminate rate varied heavily depending on the specimen bacillary load suggests that the increased sensitivity of Truenat MTB Plus to detect MTB is likely higher than that of the Truenat MTB-RIF Dx chip to detect RIF resistance, thereby producing a high number of indeterminate RIF resistance results.

The high rate of nondeterminate results seen at specific sites and by specific operators highlights the importance of appropriate on-site training, robust quality assurance/quality control programmes and effective remote monitoring. For the Truenat assays, Molbio Diagnostics’ integrated online/SIM connectivity systems can facilitate remote monitoring. In addition, it is not uncommon for nondeterminate results to be higher than normal when a new system is introduced, with improvements seen as operators gain experience with the systems. A recent study found technicians reporting comfort with assay operations after a median of 10 tests, with an associate reduction in invalid test results [26]. In terms of patient-important outcomes, quicker turnaround from testing to treatment can be expected when testing is conducted at primary healthcare centres. Overall, the Truenat assays have been estimated to be cost-effective in India compared with microscopy and Xpert [27].

Strengths of this study include the rigorous methodology employed, the use of a robust reference standard, large sample size, and the direct head-to-head comparison with Xpert MTB/RIF and Ultra. The study provides an important assessment of molecular TB test diagnostic accuracy in diverse populations representative of the global TB epidemic. However, the difficulty of diagnosing TB in real-world populations contributed to some of the limitations of the study. For example, the number of both HIV-infected participants and RIF-resistant TB cases was small, particularly so for samples tested in the primary healthcare centres, resulting in imprecise estimates of sensitivity in these groups. A recent analytical study using well-characterised *M. tuberculosis* strains showed that Truenat MTB-RIF Dx detected RIF resistance mutations representing 98.6% accuracy when weighted for global prevalence. Nevertheless, more work is needed to evaluate RIF resistance coverage in clinical settings across different geographies and patient populations [28]. Given the clear benefit of rapid diagnosis of TB in people living with HIV, further studies will be required to evaluate the accuracy of the Truenat assays in these vulnerable populations in the primary healthcare setting, particularly given the lower than anticipated performance of the Truenat assays in smear-negative culture-positive TB cases. In addition, availability issues meant that only the sites in Peru used Ultra assays, resulting in a small sample size and wider confidence intervals for the assessment of Truenat performance *versus* Ultra. Furthermore, while the heterogeneity of sputa from the same participant was controlled for by pooling sputa on day 1, use of the pooled sputa in the reference laboratory assessments could have artificially increased detection of *M. tuberculosis* in culture and Xpert *versus* Truenat assessments in the primary healthcare centre. Also, the microbiological reference standard is not perfect and may contribute to false-negative results through lengthy specimen transport or overly harsh decontamination of specimens, whereas additional diagnoses could have been made through clinical diagnosis [29]. However, culture can be standardised and is recommended by the World Health Organization (WHO) as a reference standard for evaluation of novel sputum-based diagnostics [30]. Finally, the controlled environment of this study may have contributed to evaluation conditions atypical of routine clinical operating procedures and more pragmatic studies could aid to confirm these study results.

In conclusion, this prospective clinical study demonstrates overall good performance of the Truenat assays in providing rapid diagnosis of TB and RIF resistance in intended settings of use. These results indicate that the Truenat MTB, MTB Plus and MTB-RIF Dx assays have similar accuracy to that of Xpert MTB/RIF and can be performed at the primary healthcare centre level, although data were limited for the MTB-RIF Dx assay. Findings from the Truenat assays have been reviewed by the WHO, and meet the minimal criteria for recommendation for use as an initial test for detection of TB and RIF resistance rather than smear microscopy, culture and phenotypic DST [23].

## Supplementary material

10.1183/13993003.00526-2021.Supp1 **Please note:** supplementary material is not edited by the Editorial Office, and is uploaded as it has been supplied by the author.
Supplementary material ERJ-00526-2021.Supplement

## Shareable PDF

10.1183/13993003.00526-2021.Shareable1 This one-page PDF can be shared freely online.
Shareable PDF ERJ-00526-2021.Shareable
