# Supplementary material for: A prospective multicentre diagnostic accuracy study for the Truenat tuberculosis assays
Source: Eur Respir J. 2021 Nov 4;58(5):2100526. doi: 10.1183/13993003.00526-2021 (PMC8607906; doi:10.1183/13993003.00526-2021)
Supplement: Supplementary file 1 [file ERJ-00526-2021.Supplement.pdf]

Supplementary materials

Supplementary methods

Objectives:

The two primary objectives were; firstly, to estimate the diagnostic accuracy of the Truenat assays (MTB and MTB Plus) for M. tuberculosis detection among individuals undergoing evaluation for pulmonary TB at a primary health care centre using a culture reference standard; and secondly to estimate the diagnostic accuracy of the Truenat MTB-RIF Dx assay for RIF resistance detection among individuals undergoing evaluation for pulmonary and drug-resistant TB, using phenotypic DST as the reference standard. A secondary objective was to compare the diagnostic accuracy of the Truenat assays to that of Xpert MTB/RIF and Ultra, using a reference standard of culture for TB diagnosis and phenotypic DST for detection of RIF resistance.

Procedures

Participants were enrolled at clinics at each primary health care centre. All enrolled participants had their medical history reviewed. HIV testing was offered to all participants. Participants enrolled into one of two groups, a "Case Detection Group" for those without any prior treatment for TB in the last 60 days, or a "Drug-Resistant Risk Group" for those deemed at risk of drug-resistant TB through prior failed treatment or other programmatic factors. For the Case Detection Group, all specimens were collected before initiation of TB treatment.

On Day 1, each participant was asked to submit two spot sputa of at least 2ml. Participants were given a labelled sputum cup and instructions for use, and asked to collect an additional sputum specimen (S3) the next morning (Day 2) before going to the clinic. At the clinic, participants were asked to provide a final spot sputum (S4). In the event that a participant failed to return on Day 2, S3 and S4 were permitted to be collected a maximum of 7 days after enrolment, provided that no TB treatment had been initiated (Case Detection Group).

Day 1: S1 and S2 – Two spot sputa were collected approximately 30–60 minutes apart. A smear of each sputum specimen was prepared. Thereafter, sputa totalling 4 mL or more were pooled and homogenized by glass beads and vortexing in the reference labs. This enabled comparable testing across the different index and reference tests in the centralized reference laboratory. Homogenized sputa were further split: 1·5 mL was used for analysis on raw/direct sputa, and at least 2 mL used for NALC-NaOH decontamination. Briefly, DNA was extracted independently from raw sputum and decontaminated pellet by the Trueprep Auto device and tested on both the Truenat MTB and the MTB Plus chips, both of which were read by the Truelab real-time PCR analyser. All DNA extracts testing positive by the MTB assay were subsequently tested by the Truenat MTB-RIF Dx assay (reflex), which was also read by Truelab analyser. Xpert assays were performed on the same raw and decontaminated specimens. GeneXpert systems were used for routine and study-specific Xpert testing according to manufacturer's instructions on direct sputum and decontaminated pellet [1, 2]. Mycobacteria growth indicator tube (MGIT) and Löwenstein–Jensen (LJ) culture were performed only on the decontaminated specimen. Each positive culture was identified for *M. tuberculosis* complex using MPT64 identification test. MGIT SIRE was used to determine the phenotypic DST for rifampicin (RIF).

Day 2: S3 – Morning sputum was returned to the clinic in a labelled sputum cup. S3 was sent to the reference laboratory and a second round of MGIT and LJ culture was performed on the decontaminated sediment.

Day 2: S4 – At the time that S3 was returned to the clinic, the participant was asked to provide spot sputum S4. The intended objective of this additional sputum specimen was to test the Truenat assay in the setting of use (i.e. primary health care centres with associated microscopy centres). In sites where the primary health care centre and the reference lab are the same, the Truenat assays were only performed once alongside Xpert (on Day 1). As PD Hinduja Hospital in India and the Port Moresby General Hospital in Papua New Guinea are centralized laboratory facilities, Sputum 4 was not collected as no dedicated microscopy centre as part of a primary health care centre was available. Spot sputum S4 was processed in the primary health care centre: the entire volume of sputum was liquefied and lysed using Trueprep Auto kit reagents, and 500 µL raw sputum was used for DNA extraction by Trueprep Auto and MTB detection by the Truenat assays. Any *M. tuberculosis*-positive specimens were subsequently tested by the reflex Truenat MTB-RIF Dx assay.

All positive Truenat chips were stored (refrigerated) at the sites to allow for sequencing from DNA amplicons if required for discordance resolution, as pre-defined in the protocol. Additionally, any leftover sputum, pellet, and culture isolates positive for non-tuberculous mycobacteria or *M. tuberculosis* were stored (frozen).

For all sample testing days, quality control was conducted through daily negative control testing (sterile water across all lysis, extraction and PCR steps) and weekly swab testing of workspace, external equipment and internal PCR trays on Truenat MTB Plus chips.

Staff performing either the index test of the reference standard were blinded to results of other study tests through the use of specimen codes and staffing assignments. Data were captured through dedicated data-entry systems that were password protected.

Sample size calculation

For the primary analyses, a sample size of 1,666 participants was selected to allow analysis of 80 smear-negative culture-positive TB cases across sites (95% CI: 55, 77), based on an estimated 67% Truenat MTB Plus sensitivity, a TB prevalence of 20%, and a 30% prevalence of smear-negative, culture-positive TB cases. We estimated 2.8% RIF resistance among all culture-positive TB cases, and 12% prevalence of RIF resistance amongst TB retreatment cases. PD Hinduja Hospital, a DR-TB referral centre in Mumbai, India, was specifically selected to increase enrolment of participants into the Drug-Resistance Risk Group.

Analysis

Participants in the Case Detection Group were included in all analyses, whereas participants in the Drug-Resistant Risk Group were only included in analyses of rifampicin-resistance detection.

Case definitions for primary analyses were as follows:

- The reference standard for TB classification was based on TB culture and *M. tuberculosis* complex (MTBC) identification results: a specimen was defined as TB positive if at least one of the culture results was positive and confirmed MTBC; a specimen was defined as negative if no culture was positive for MTBC and at least two culture results were negative. A TB case was defined as one with any TB-positive specimen.
- For RIF detection, the analyses were based on phenotypic DST results.
- Smear-positive, culture negative specimens were excluded.

Analyses of the diagnostic accuracy of the Truenat index tests and comparator tests were conducted per case or per specimen in the Case Detection Group and reported as point estimates and 95% confidence intervals based on Wilson's score method. Subgroup analyses by site of testing (microscopy centre versus reference laboratory for Truenat), by smear status, TB history and HIV status were performed.

The proportion of non-determinate results, defined as any non-valid results, was assessed in both clinics and reference laboratories. These non-determinate results included both operator errors and equipment/software errors or failures, or invalid results or indeterminate results.

The study protocol and statistical analysis plan are available in the supplementary materials. All statistical analysis was performed using R version 3.5.1.

Supplementary results:

Quality control

Positive results from testing swabs and negative controls were rare, indicating appropriate daily cleaning and handling of materials. All positive results were resolved after cleaning and did not persist or inhibit subsequent specimen testing. Days where swabs or negative controls tested positive never coincided with days where participant specimens tested false-positive, suggesting that the risk of carry-over contamination was low in the context of this study.

Performance in participants with a history of TB

In a sub-analysis of all patients with and without a history of TB disease, the specificity of all Truenat assays was lower in participants with a history of TB disease, as seen for Xpert MTB/RIF and Ultra (Supplementary Table 8).

Discordance analysis

Overall, 126 participants had at least one false-positive result and 131 participants had at least one false-negative result by either Truenat MTB or MTB Plus assays on at least one of the six tests done per participant. Of the 126 participants with false-positive results, 40 were also false-positive by either Xpert MTB/RIF or Ultra. None of the false-positive results coincided with positive test results on negative controls or swabs.

Supplementary references

1. Cepheid. Xpert® MTB/RIF package insert 2020 [Internet]. Available from: https://www.cepheid.com/Package%20Insert%20Files/Xpert-MTB-RIF-ENGLISH-Package-Insert-301-1404-Rev-F.pdf.

2. Cepheid. Xpert® MTB/RIF Ultra 2020 [Internet]. Available from: https://www.cepheid.com/en/tests/Critical-Infectious-Diseases/Xpert-MTB-RIF-Ultra.

Supplementary Table S1. Participating sites

**India**	Site 01	Mumbai: Hinduja
**India**	Site 02	Guwahati: Kamrup
	Site 03	Guwahati: Railway
	Site 04	Guwahati: Sonapur
	Ref Lab	Guwahati: Intermediate Reference Laboratory, Guwahati Medical College
**India**	Site 05	Chennai: Ayanavaram
	Site 06	Chennai: Villiwakkam
	Site 07	Chennai: Thanthai Perivar
	Ref Lab	Chennai: National Institute of Research in Tuberculosis
**India**	Site 08	Ahmedabad: Madhupura
	Site 09	Ahmedabad: CHC Chhala
	Site 10	Ahmedabad: PHC Kuha
	Ref Lab	Ahmedabad: Intermediate Reference Laboratory, State TB and Demonstration Center, Civil Hospital Campus
**Peru**	Site 11	Lima: CS Huascar II
	Site 12	Lima: CS Huascar XV
	Site 13	Lima: CS Jose Carlos Mariategui
	Site 14	Lima: CS Fraternidad
	Site 19	Lima: CS El Porvenir
	Ref Lab	Lima: Universidad Peruana Cayetano Heredia
**Ethiopia**	Site 15	Addis Ababa: Hiwot Amba
	Site 16	Addis Ababa: St. Gebrel
	Site 17	Addis Ababa: Woreda 01
	Ref Lab	Addis Ababa: Ethiopian Public Health Institute
**Papua New Guinea**	Site 18	Port Moresby: Central Public Health Laboratory, Port Moresby General Hospital

Supplementary Table S2. Participant inclusion and exclusion criteria for the Case Detection and Drug-Resistant Risk TB Groups

Case Detection Group	Drug-Resistant Risk Group
**Inclusion criteria**	
Age 18 years or above<br>Provision of informed consent<br>Willingness to provide at least 3 sputum specimens (>2 mL) at enrolment	
• Willingness to have a study follow-up visit approximately 42 to 70 days after enrolment<br>• Clinical suspicion of pulmonary TB (including cough ≥2 weeks and at least 1 other symptom typical of TB)	• Non-converting pulmonary TB cases (category I and category II failures)<br>• Retreatment cases* (those having failed a regimen, relapses or returned after loss to follow-up)<br>• Close contacts of drug-resistant TB patients who have been diagnosed with active TB*<br>• Participants at high risk for MDR-TB as determined by local programme*
**Exclusion criteria**	
• Receipt of any dose of TB treatment within 60 days prior to enrolment<br>• Participants for whom, at the time of enrolment, the follow-up visit was poorly feasible (e.g. individuals planning to relocate)	• Receipt of any MDR-TB treatment within 60 days prior to enrolment

MDR-TB, multi-drug resistant tuberculosis; TB, tuberculosis.

*Pulmonary TB cases on TB treatment were eligible if they were suspected to be treatment failures irrespective of how long TB treatment had been ongoing. All culture-negative study participants on TB treatment were excluded from the analysis, even if they were smear-positive.

Supplementary Table S3. Reference standard test and index test procedures

Test	Notes*
**Smear**	All sites used light microscopy (Ziehl Neelsen), except for PD Hinduja Hospital which used fluorescence microscopy (auramine-O), and sites in Peru, which used both methods. Testing and reporting was undertaken as per WHO/IUATLD guidelines (1).
**Xpert MTB/RIF**	2:1 sample reagent added to raw sputum. In case of invalid, error or no result, testing was repeated if enough specimen was available.
**Ultra**	2:1 sample reagent added to raw sputum and pellet (2). In case of invalid, error or no result, testing was repeated if enough specimen was available.
**Liquid culture**	Mycobacteria Growth Indicator Tube 960 culture; BD Microbiology Systems
**Solid culture**	Löwenstein Jensen. Testing and reporting done as per GLI mycobacteriology laboratory manual and local guidelines.
**MGIT DST**	BD MGIT AST SIRE Test kit
**MTB identification**	MPT-64, SD Bioline, BD, or Capilia TB-Neo, TAUNS

IUATLD, International Union Against Tuberculosis and Lung Disease; WHO, World Health Organization.
*Testing done as per manufacturer's instructions unless otherwise specified.

Supplementary Table S4. Performance of Truenat assays for TB and for RIF resistance detection at the primary health care centre (microscopy centre) and the reference laboratory, for participants with result for either Truenat MTB, MTB Plus or MTB-RIF Dx assays.

All participants	N	TP	FP	FN	TN	Sensitivity % (95% CI)	Sensitivity % Smear Pos (95% CI) - N	Sensitivity % Smear Neg (95% CI) - N	Specificity % (95% CI)
**Microscopy Centre sputum**									
Truenat MTB	1402	192	25	71	1114	73.0 [67.3,78.0]	91.0 [85.8,94.4] - N:177	36.0 [26.7,46.6] - N:86	97.8 [96.8,98.5]
Truenat MTB Plus	1369	212	41	54	1062	79.7 [74.5,84.1]	96.1 [92.2,98.1] - N:179	46.0 [35.9,56.4] - N:87	96.3 [95.0,97.2]
Truenat MTB Rif-Dx	190	16	9	3	162	84.2 [62.4,94.5]	87.5 [64.0,96.5] - N:16	66.7 [20.8,93.8] - N:3	94.7 [90.3,97.2]
**Reference lab sputum**									
Truenat MTB	1603	278	28	74	1223	79.0 [74.4,82.9]	95.8 [92.4,97.7] - N:238	43.9 [35.1,53.0] - N:114	97.8 [96.8,98.5]
Truenat MTB Plus	1552	297	52	51	1152	85.3 [81.2,88.7]	98.3 [95.7,99.3] - N:236	58.0 [48.8,66.8] - N:112	95.7 [94.4,96.7]
Truenat MTB Rif-Dx	332	44	9	8	271	84.6 [72.5,92.0]	86.7 [73.8,93.7] - N:45	71.4 [35.9,91.8] - N:7	96.8 [94.0,98.3]

FN, false negative; FP, false positive; TN, true negative; TP, true positive.

Note: Analysis of Truenat performance is shown on specimens collected at the microscopy centre and at the reference laboratory separately, with valid results available for either the Truenat MTB assay or the Truenat MTB Plus assay; denominators differ based on the number of valid results for each assays, and as two sites (PD Hinduja hospital and Papua New Guinea) only had reference lab facilities available.

Supplementary Table S5. Performance of the Truenat assays performed in primary health care centres (microscopy centres) and reference laboratories

	N	TP	FP	FN	TN	Sensitivity % (95% CI)	Sensitivity % Smear Pos (95% CI) - N	Sensitivity % Smear Neg (95% CI) - N	Specificity % (95% CI)
**Truenat MTB**									
Ref Lab sputum	1376	203	25	56	1092	78.4 [73.0,83.0]	97.1 [93.5,98.8] - N:175	39.3 [29.5,50.0] - N:84	97.8 [96.7,98.5]
Microscopy Centre sputum	1376	189	24	70	1093	73.0 [67.3,78.0]	90.9 [85.7,94.3] - N:175	35.7 [26.3,46.4] - N:84	97.9 [96.8,98.6]
Difference (Microscopy Centre - Ref lab)						-5.4 [-10.0,-1.2]	-6.2 [-11.3,-2.3]	-3.6 [-13.8,+6.5]	+0.1 [-0.9,+1.1]
**Truenat MTB Plus**									
Ref Lab sputum	1311	215	43	44	1009	83.0 [78.0,87.1]	98.3 [95.1,99.4] - N:176	50.6 [40.1,61.1] - N:83	95.9 [94.5,97.0]
Microscopy Centre sputum	1311	208	39	51	1013	80.3 [75.0,84.7]	96.6 [92.8,98.4] - N:176	45.8 [35.5,56.5] - N:83	96.3 [95.0,97.3]
Difference (Microscopy Centre - Ref lab)						-2.7 [-7.1,+1.5]	-1.7 [-5.5,+1.6]	-4.8 [-16.3,+6.6]	+0.4 [-1.0,+1.8]
**Truenat RIF**									
Ref Lab sputum	175	14	7	3	151	82.4 [59.0,93.8]	81.2 [57.0,93.4] - N:16	100 [20.6,100.0] - N:1	95.6 [91.1,97.8]
Microscopy Centre sputum	175	15	9	2	149	88.2 [65.7,96.7]	87.5 [64.0,96.5] - N:16	100 [20.6,100.0] - N:1	94.3 [89.5,97.0]
Difference (Microscopy Centre - Ref lab)						+5.8 [-13.6,+27.0]	+6.3 [-14.3,+28.3]	0 [-79.3,+79.3]	-1.3 [-4.9,+1.8]

Note: Differences in sensitivity and specificity were calculated as performance of each Truenat assay conducted in the microscopy centre (Day 2) minus that conducted in the reference lab (Day 1), relative to *M. tuberculosis* culture (for TB detection) or RIF DST (for RIF resistance detection).

Supplementary Table S6. Performance of the Truenat assays for TB and RIF resistance detection compared to Xpert MTB/RIF

		N	TP	FP	FN	TN	Sensitivity % (95% CI)	Sensitivity % Smear Pos (95% CI) - N	Sensitivity % Smear Neg (95% CI) - N	Specificity % (95% CI)
Case Detection Group only	**Truenat MTB**									
	Xpert	1162	217	27	36	882	85.8 [80.9,89.5]	98.9 [96.1,99.7] - N:185	50.0 [38.4,61.6] - N:68	97.0 [95.7,98.0]
	Truenat MTB	1162	208	25	45	884	82.2 [77.0,86.4]	96.2 [92.4,98.2] - N:185	44.1 [33.0,55.9] - N:68	97.2 [96.0,98.1]
	Difference (Truenat MTB- Xpert)						-3.6 [-7.8,+0.3]	-2.7 [-6.4,+0.1]	-5.9 [-18.6,+6.7]	0.2 [-0.8,+1.3]
	**Truenat MTB Plus**									
	Xpert	1162	217	27	36	882	85.8 [80.9,89.5]	98.9 [96.1,99.7] - N:185	50.0 [38.4,61.6] - N:68	97.0 [95.7,98.0]
	Truenat Plus MTB	1162	222	43	31	866	87.7 [83.1,91.2]	98.9 [96.1,99.7] - N:185	57.4 [45.5,68.4] - N:68	95.3 [93.7,96.5]
	Difference (Truenat Plus MTB - Xpert)						1.9 [-1.3,+5.6]	0 [-2.5,+2.5]	7.4 [-4.2,+19.2]	-1.7 [-3.2,-0.5]
Case Detection and DR-Risk Group	**RIF detection**									
	Xpert Rif	252	37	6	5	204	88.1 [75.0,94.8]	89.7 [76.4,95.9] - N:39	66.7 [20.8,93.8] - N:3	97.1 [93.9,98.7]
	Truenat Rif	252	35	7	7	203	83.3 [69.4,91.7]	84.6 [70.3,92.8] - N:39	66.7 [20.8,93.8] - N:3	96.7 [93.3,98.4]
	Difference (Truenat Rif - Xpert Rif)						-4.8 [-15.8,+4.0]	-5.1 [-16.9,+4.3]	0 [-56.1,+56.1]	-0.4 [-2.6,+1.3]

Note: Differences in sensitivity and specificity were calculated as performance of each Truenat assay conducted in the reference laboratory (Day 1) for Truenat assays minus Xpert MTB/RIF, on the same homogenized specimen, relative to *M. tuberculosis* culture (for TB detection) or RIF DST (for RIF resistance detection). As Peru did not run the Xpert MTB/RIF assay, this site did not contribute to the analysis shown here.

Supplementary Table S7. Performance of the Truenat assays for TB and RIF resistance detection compared to Ultra (Peru only)

		N	TP	FP	FN	TN	Sensitivity % (95% CI)	Sensitivity % Smear Pos (95% CI) - N	Sensitivity % Smear Neg (95% CI) - N	Specificity % (95% CI)
Case Detection Group only	**Truenat MTB**									
	Ultra	378	88	8	5	277	94.6 [88.0,97.7]	100.0 [93.0,100.0] - N:51	88.1 [75.0,94.8] - N:42	97.2 [94.6,98.6]
	Truenat MTB	378	67	2	26	283	72.0 [62.2,80.2]	94.1 [84.1,98.0] - N:51	45.2 [31.2,60.1] - N:42	99.3 [97.5,99.8]
	Difference (Truenat MTB - Ultra)						-22.6 [-32.1,-15.3]	-5.9 [-15.9,+1.5]	-42.9 [-57.8,-29.1]	2.1 [0.7,+4.5]
	**Truenat MTB Plus**									
	Ultra	378	88	8	5	277	94.6 [88.0,97.7]	100.0 [93.0,100.0] - N:51	88.1 [75.0,94.8] - N:42	97.2 [94.6,98.6]
	Truenat Plus MTB	378	73	7	20	278	78.5 [69.1,85.6]	96.1 [86.8,98.9] - N:51	57.1 [42.2,70.9] - N:42	97.5 [95.0,98.8]
	Difference (Truenat MTB Plus - Ultra)						-16.1 [-24.9,-10.0]	-3.9 [-13.2,+3.4]	-31 [-46.0,-19.1]	0.3 [-1.8,+2.6]
Case Detection and DR-Risk Group	**RIF detection**									
	Ultra Rif	70	7	2	0	61	100 [64.6,100.0]	100 [61.0,100.0] - N:6	100 [20.6,100.0] - N:1	96.8 [89.1,99.1]
	Truenat Rif	70	7	2	0	61	100 [64.6,100.0]	100 [61.0,100.0] - N:6	100 [20.6,100.0] - N:1	96.8 [89.1,99.1]
	Difference (Truenat Rif - - Ultra Rif)						0 [-35.4,+35.4]	0 [-39.0,+39.0]	0 [-79.3,+79.3]	0 [-5.7,+5.7]

Note: Differences in sensitivity and specificity were calculated as performance of each Truenat assay conducted in the reference laboratory (Day 1) for Truenat assays minus Ultra, on the same homogenized specimen, relative to *M. tuberculosis* culture (for TB detection) or RIF DST (for RIF resistance detection). As Peru was the only site to run the Ultra assay, no other site contributed to the analysis shown here.

Supplementary Table S8. Specificity of the Truenat assays compared with Xpert MTB/RIF and Ultra among participants with and without a prior history of TB

		All samples	Specificity % - TB History (95% CI)		Specificity % - No TB History (95% CI)	
Compared to Xpert	Case Detection Group only	**Truenat MTB**				
		Xpert MTB/RIF	93.3	[86.2,96.9]	97.3	[95.9,98.2]
		Truenat MTB	94.4	[87.6,97.6]	97.4	[96.1,98.3]
		Difference (Truenat MTB - Xpert)	+1.1	[-4.1,+6.8]	+0.1	[-1.0,+1.3]
		**Truenat MTB Plus**	--	--	--	--
		Xpert MTB/RIF	91.5	[83.4,95.8]	97.3	[95.9,98.3]
		Truenat Plus MTB	90.2	[81.9,95.0]	95.6	[93.9,96.9]
		Difference (Truenat Plus MTB - Xpert)	-1.3	[-8.9,+6.2]	-1.7	[-3.2,-0.4]
	Case Detection and DR-Risk Group	**RIF detection**				
		Xpert MTB/RIF Rif	93.8	[71.7,98.9]	97.9	[94.7,99.2]
		Truenat Rif	93.8	[71.7,98.9]	97.4	[94.0,98.9]
		Difference (Truenat Rif - Xpert Rif)	0	[-19.4,+19.4]	-0.5	[-2.9,+1.5]
Compared to Ultra	Case Detection Group only	**Truenat MTB**				
		Ultra	92.9	[85.3,96.7]	99.0	[96.5,99.7]
		Truenat MTB	97.6	[91.7,99.3]	100	[98.2,100.0]
		Difference (Truenat MTB - Ultra)	+4.7	[+0.2,+11.6]	+1.0	[-0.9,+3.5]
		**Truenat MTB Plus**	--	--	--	--
		Ultra	92.9	[85.3,96.7]	99.0	[96.5,99.7]
		Truenat Plus MTB	92.9	[85.3,96.7]	99.5	[97.3,99.9]
		Difference (Truenat Plus MTB - Ultra)	0	[-6.2,+6.2]	+0.5	[-1.8,+3.1]
	Case Detection and DR-Risk Group	**RIF detection**				
		Ultra Rif	100	[67.6,100.0]	96.4	[87.7,99.0]
		Truenat Rif	100	[67.6,100.0]	96.4	[87.7,99.0]
		Difference (Truenat Rif - Ultra)	0	[-32.4,+32.4]	0	[-6.5,+6.5]

Note: Differences in sensitivity and specificity were calculated as performance of each Truenat assay minus Xpert MTB/RIF or minus Ultra for the reference lab sputum relative to MTB culture (for TB detection). Only participants in the Case Detection Group were included in the TB detection analyses for Truenat MTB and Truenat MTB Plus. Truenat performance comparisons were drawn against Xpert MTB/RIF at all sites except Peru, where Ultra was performed as the comparator.

Supplementary Table S9. Proportion of non-determinate assay results for Trueprep extraction, Truenat assays and Xpert MTB/RIF and Ultra assays

Total non-determinates	Initial Test		Repeat Test	
	(%)	n/N	(%)	n/N
Trueprep	2.4%	113/4732	11.7%	13/111
Truenat MTB	6.2%	293/4720	21.2%	62/293
Truenat MTB Plus	9.2%	434/4720	36.8%	159/432
Truenat MTB RIF-Dx*	22.5%	232/1042	72.7%	157/216
Xpert MTB/RIF	2.6%	65/2522	7.9%	5/63
Xpert Ultra	0.0%	0/786	-	-

*Truenat MTB-RIF Dx was run on any specimen that tested positive for *M. tuberculosis* by either the Truenat MTB assay or the Truenat MTB Plus assay.

Note: Data represents all assays run, not the number of participants with any non-determinate result. Non-determinate results represent a combination of operator and equipment errors or failures, invalid results and indeterminate results, for all participant specimens tested as part of this study. The non-determinate results for the Truelab micro PCR machine represent results for all different Truenat assay performed at each site. Not all specimens that failed on the initial test were still available for repeat testing. The results presented here do not capture errors in DNA loading or chip loading as site incident logs did not report high levels of such errors.

Supplementary Table S10. The proportion of non-determinate Truenat MTB-RIF Dx results when reflexed from either the Truenat MTB or MTB Plus TB detection result

	Truenat RIF-Dx Non-determinates	
	% (95% CI)	n/N
If reflexed from Truenat MTB-pos and MTB Plus-pos	3.9% (2.7, 5.4)	32/830
If reflexed from Truenat MTB-neg and MTB Plus-pos	67% (60, 74)	120/179
If reflexed from Truenat MTB-pos and MTB Plus-neg	72% (56, 84)	26/36
If reflexed only from Truenat MTB-pos	6.7% (5.2, 8.6)	58/866
If reflexed only from Truenat MTB-Plus-pos	15% (13, 17)	152/1009

Supplementary Figure S1. Proportion of participants with non-determinate Truenat assay results at initial testing and after repeat testing, stratified by sputum sample

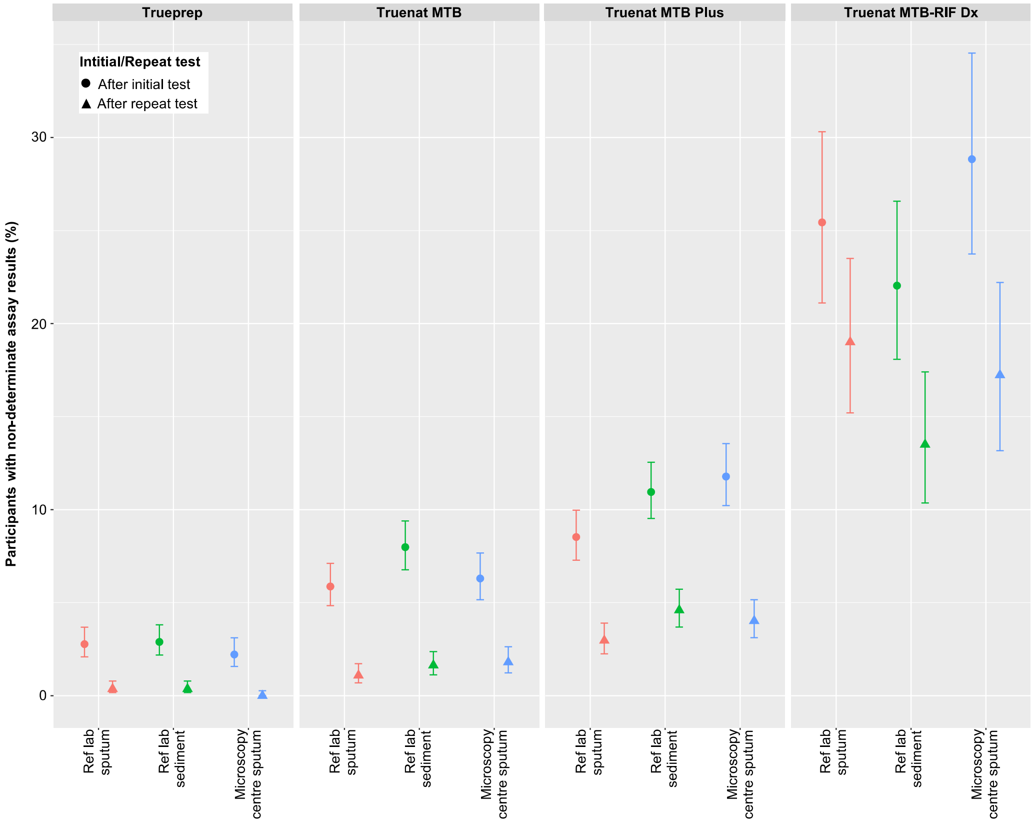

Note: Data represents the proportion of enrolled participants with a non-determinate Truenat assay result when run as an initial test and, if required, when repeated. Non-determinate results represent a combination of operator and equipment errors or failures, invalid results and indeterminate results, for all participant specimens tested as part of this study. Not all specimens that failed on the initial test were still available for repeat testing. Samples were only reflexed to the Truenat MTB-RIF Dx assay if a positive test for MTB detection was reported on either the Truenat MTB or Truenat MTB Plus chip.
