# Supplementary material for: A prospective multicentre diagnostic accuracy study for the Truenat tuberculosis assays
Source: Eur Respir J. 2021 Nov 4;58(5):2100526. doi: 10.1183/13993003.00526-2021 (PMC8607906; doi:10.1183/13993003.00526-2021)
Supplement: Supplementary file 2 [file ERJ-00526-2021.Shareable.pdf]

SHAREABLE PDF

A prospective multicentre diagnostic accuracy study for the Truenat tuberculosis assays

Adam Penn-Nicholson[1], Sivaramakrishnan N. Gomathi[2], Cesar Ugarte-Gil[3,4], Abyot Meaza[5], Evelyn Lavu[6], Pranav Patel[7], Bandana Choudhury[8], Camilla Rodrigues[9], Sarabjit Chadha[10], Mubin Kazi[9], Aurélien Macé[1], Pamela Nabeta[1], Catharina Boehme[1], Raman R. Gangakhedkar[11], Sanjay Sarin[10], Ephrem Tesfaye[5], Eduardo Gotuzzo[3], Philipp du Cros[12], Srikanth Tripathy[2], Morten Ruhwald[1], Manjula Singh[11], Claudia M. Denkinger[1,13,14], Samuel G. Schumacher[1,14] and the Truenat Trial Consortium[15]

[1]FIND, Geneva, Switzerland. [2]National Institute for Research in Tuberculosis, Chennai, India. [3]Instituto de Medicina Tropical Alexander von Humboldt, Lima, Peru. [4]School of Medicine, Universidad Peruana Cayetano Heredia, Lima, Peru. [5]Ethiopian Public Health Institute, Addis Ababa, Ethiopia. [6]Central Public Health Laboratory, Port Moresby, Papua New Guinea. [7]State TB Demonstration and Training Centre, Ahmedabad, India. [8]Intermediate Reference Laboratory, Guwahati, India. [9]PD Hinduja Hospital, Mumbai, India. [10]FIND – India, New Delhi, India. [11]Indian Council of Medical Research, New Delhi, India. [12]Burnet Institute, Melbourne, Australia. [13]Division of Tropical Medicine, Center of Infectious Disease, University Hospital Heidelberg, Heidelberg, Germany. [14]These authors contributed equally. [15]The members of the Truenat Trial Consortium are listed in the Acknowledgements.

Corresponding author: Adam Penn-Nicholson (adam.penn-nicholson@finddx.org)

Check for updates

Shareable abstract (@ERSpublications)
Diagnostic performance of point-of-care Truenat assays in primary healthcare centres is comparable to that of Xpert MTB/RIF placed in reference laboratories. The WHO now recommends Truenat as an initial test for detection of TB and RIF resistance. https://bit.ly/31Wj3S6

Cite this article as: Penn-Nicholson A, Gomathi SN, Ugarte-Gil C, *et al.* A prospective multicentre diagnostic accuracy study for the Truenat tuberculosis assays. *Eur Respir J* 2021; 58: 2100526 [DOI: 10.1183/13993003.00526-2021].

This single-page version can be shared freely online.

Copyright ©The authors 2021.

This version is distributed under the terms of the Creative Commons Attribution Licence 4.0.

This article has supplementary material available from erj.ersjournals.com

Received: 19 Feb 2021
Accepted: 29 March 2021

Abstract

Background Bringing reliable and accurate tuberculosis (TB) diagnosis closer to patients is a key priority for global TB control. Molbio Diagnostics have developed the Truenat point-of-care molecular assays for detection of TB and rifampicin (RIF) resistance.

Methods We conducted a prospective multicentre diagnostic accuracy study at 19 primary healthcare centres and seven reference laboratories in Peru, India, Ethiopia and Papua New Guinea to estimate the diagnostic accuracy of the point-of-care Truenat MTB, MTB Plus and MTB-RIF Dx assays for pulmonary TB using culture and phenotypic drug susceptibility testing as the reference standard, compared with Xpert MTB/RIF or Ultra.

Results Of 1807 enrolled participants with TB signs/symptoms, 24% were culture-positive for *Mycobacterium tuberculosis*, of which 15% were RIF-resistant. In microscopy centres, the pooled sensitivity of Truenat MTB and Truenat MTB Plus was 73% (95% CI 67–78%) and 80% (95% CI 75–84%), respectively. Among smear-negative specimens, sensitivities were 36% (95% CI 27–47%) and 47% (95% CI 37–58%), respectively. Sensitivity of Truenat MTB-RIF was 84% (95% CI 62–95%). Truenat assays showed high specificity. Head-to-head comparison in the central reference laboratories suggested that the Truenat assays have similar performance to Xpert MTB/RIF.

Conclusion We found the performance of Molbio's Truenat MTB, MTB Plus and MTB-RIF Dx assays to be comparable to that of the Xpert MTB/RIF assay. Performing the Truenat tests in primary healthcare centres with very limited infrastructure was feasible. These data supported the development of a World Health Organization policy recommendation of the Molbio assays.
